# Dental Fistula

**Published:** 1883-01

**Authors:** Chevalier Cacciaouerra, C. W. Dunn

**Affiliations:** Catania, Italy.; Florence.


### ARTICLE VI.

### *On Dental Fistula.*

BY CHEVALIER CACCIAOUERRA, M. D., OF CATANIA, ITALY.

[Translated from the Italian by C. W. Dunn, L D. S. Eng., of Florence.]

Through the effect of caries, either in the second or third stage, in subjects affected by constitutional tendency, under the influence of occasional causes, inflammation of the dental pulp and periosteum often occurs, causing a swelling, accompanied by inflammation, extending to all the maxillary region, which may terminate either in absorption or suppuration ; the anti-inflammatory treatment facilitiates these two results. In absorption all is finished, whereas in suppuration, when every other inflammatory symptom has ceased, there often remains an alveolar abscess and fistula, produced sometimes by the purulent secretion of caries, sometimes by the introduction of air, which passing through the dental canals comes into contact with the periosteum and alveolar membrane. These becoming inflamed communicative the inflammation to the external tissue of the gums on the edge of the alveolar process, which is the point it generally selects ; there may also be developed an irritation solely of the soft parts of the dental canals, with or without a slight inflammation on the edge of the external maxillary bone.

The abscess has almost the form of half a pea, and contains a secretion either watery, or of matter, or air or blood, according to the predominant tendency in the system of the patient. The spontaneous or artificial discharge of the contents cause pain to cease, and diminishes the partial inflammation (if such exists), both secondary symptoms depending on the caries, which with the gradual inflammatory process it generates, destroys the pulp and with it the abscess.

In order to cure this disease, and not wishing to extract the tooth, I made a transverse puncture, which, facilating the discharge of the contents of the abscess, brought abont diminution of volume during recovery, and little by little the cure; this happening after some time had passed, I was in doubt as to whether it was the effect of the treatment or simply the effect of the disease itself which had destroyed the dental pulp; nevertheless I used this method, as by it, I obtained restriction of the disease until a complete curve was affected.

More than a year after the replanting of the first bicuspid on the left side of the upper jaw, contrary to the usual course, the dental fistula was not cured by the grafting, but only diminished in volume and in extent. Hoping for a cure with time, I allowed some months to pass, but the abscess was persistent, being kept up by the second bicuspid, which was also decayed, and which had been filled before the grafting of the first. Instead of making a second replantation I wished to try the effect of chromic acid.

With a straight pair of scissors I removed the projecting gum round the fistula, and there remained then exposed a wound of circular form, to which I applied chromic acid on lint, leaving it for twenty-four hours; the next day I removed the dressing, and with it the crust. I then renewed the cauterization, leaving the lint for an hour only on the wound. Similar treatment was repeated for three days, and I obtained the destruction of the base of the tissue which formed the abscess. I then left the last crust to fall off by itself, and in nine days the cure was complete.

Having succeeded in this first case, I wished to try it in others, where the tooth had not just before undergone the operation of grafting, and so to prove whether in all dental abscesses I could attain the same success, and I had seven other successful cases, in the course of which I found that if the inflammation around the wound had not entirely ceased, and although no secretion might be formed, the application of chromic acid ought to be renewed every day or two.

The result of this method is the total destruction of the dental abscess, leaving uncovered that portion of the root which sustained it. In some cases different degrees of sensitiveness when in contact with external agents remain, in others insensibility.

The circular border which surrounds the denuded part presents a healty color, which in progress of time gradually closes without producing any irritation, and for two reasons: the first, because after the total destruction of the tissues and of the periosteum of the root, a species of atrophy takes place in the denuded part, forming a layer of very fine dentine; secondly, because in the parts where the abscess was formed there are no longer any soft parts to become inflamed.—*British Journal of Dental Science.*

---

